# Navigating challenges in medical english learning: leveraging technology and gamification for interactive education – a qualitative study

**DOI:** 10.1186/s12909-025-07511-1

**Published:** 2025-07-12

**Authors:** Zahra Zolfaghari, Zahra Karimian, Nahid Zarifsanaiey, Amir Yousef Farahmandi

**Affiliations:** 1 Department of E-Learning in Medical Sciences, Virtual School and Center of Excellence in e-Learning, Student Research Committee, Shiraz University of Medical Sciences, Shiraz, Iran; 2 Department of E-Learning in Medical Sciences, Virtual School and Center of Excellence in e-Learning, Shiraz University of Medical Sciences, Shiraz, Iran; 3 Department of E-learning, Virtual School, Center of Excellence for e-Learning in Medical Sciences, Shiraz University of Medical Sciences, Shiraz, Iran; 4 Department of English Language, School of Paramedical Sciences, Foreign Language, Shiraz University of Medical Sciences, Shiraz, Iran

**Keywords:** Medical english, English for medical purposes, Medical education, Technology integration, Gamification

## Abstract

**Background:**

Teaching and learning English for Medical Purposes is crucial for medical students, especially in non-English-speaking countries, as it facilitates effective communication, access to global medical literature, and participation in international medical events. However, medical English education faces numerous challenges. This qualitative study explores the challenges faced by medical educators and students in Iran and proposes strategies to enhance medical English education through technology and gamification.

**Method:**

A thematic content analysis was conducted using semi-structured interviews with 13 medical English instructors and 10 medical students from various Iranian universities. Data were collected from September 2023 to February 2024. The interviews focused on challenges in teaching and learning medical English, the role of technology, and potential improvements. Thematic analysis was employed to identify key themes and sub-themes.

**Results:**

The study identified six main themes: (1) Instructor-related challenges (e.g., knowledge gaps, teaching methodology, and resistance to technology), (2) Student-related challenges (e.g., low language proficiency, pronunciation difficulties, and lack of engagement), (3) Curriculum and content issues (e.g., outdated materials, lack of speaking practice), (4) Organizational challenges (e.g., unclear policies, resistance to technological change), (5) Technology-enhanced learning (e.g., inadequate infrastructure, lack of interactive platforms), and (6) Recommendations for improvement (e.g., joint teaching, gamification, and blended learning).

**Conclusion:**

The study indicated the need for a comprehensive approach to medical English education in Iran, emphasizing instructor training, interactive learning, updated curricula, and technology integration. By addressing these challenges, medical English education can be significantly improved, allowing future physicians to become proficient in linguistic skills which improves the globalized healthcare environment.

**Supplementary Information:**

The online version contains supplementary material available at 10.1186/s12909-025-07511-1.

## Introduction

Teaching and learning English for Medical Purposes (EMP) are essential features in medical education, particularly in non-English-speaking countries [1]. To become future doctors, medical students must learn medical knowledge and master English to communicate effectively, which is often the common language for medical literature and professional purposes [2]. Besides, most medical events, conferences, workshops, and collaborative research projects are usually held in English [3, 4].

So, proficiency in English enables medical students to connect with peers and experts and leads to collaboration and innovation in medical fields [5]. Also, in many countries, healthcare environments host diverse populations that may include English-speaking patients [6]. Effective communication is necessary to perceive patient histories [7], explain diagnoses [8], and discuss treatment options [9]. Proficiency in English improves the ability to provide high-quality care and patient safety. So, by mastering English, future physicians can reduce these risks and ensure clearer communication with patients and colleagues [10].

Combining English language training into medical schools’ curricula offers unique challenges that can interfere with the learning experience [10, 11]. Previous research has highlighted various obstacles instructors experience while teaching specialized English, including a lack of familiarity with medical terminology and insufficient student engagement [12, 13]. Similarly, students often struggle with pronunciation, comprehension, and specialized vocabulary usage in clinical contexts [14]. A study by Hani Elgindi and colleagues analyzed the challenges faced by novice teachers of EMP in Saudi Arabia’s Medical Colleges. It revealed that new teachers faced difficulties adjusting English words to the medical context, pronunciation, and understanding medical terminology. They encountered challenges with English used in medical procedures, daily routines in healthcare settings, and communication with medical staff and patients and there was little support available to help them cope with these issues. Additionally, pedagogical concerns, particularly regarding methodology, posed further challenges for these educators. The findings highlighted the need for solutions to improve the performance of teachers and their students [15]. Another study, published by Miao Yang and colleagues in China, revealed that implementing EMP instruction is challenging as it necessitates faculty development, institutional backing, and effective self and peer-learning strategies among students. So, the adaptive strategies utilized by students and instructors can provide valuable insights for enhancing the implementation of programs [16].

A study on English for Specific Purposes (ESP) teachers indicated that while students were generally satisfied with their instructors, several important aspects of ESP courses were often neglected. Key areas that needed attention included addressing students’ needs, introducing relevant discourse communities, practicing appropriate tasks, and providing supplementary and authentic materials. Students also faced challenges such as limited class time, uneven credit hour distribution, and varying skill levels within classes, which interfered with the achievement of course objectives. To overcome these challenges, the study emphasized the need for improved course design, effective technology use, communicative approaches, and better time management in ESP classes [12].

Dr. Farida Djaileb conducted a study on the challenges of teaching ESP in Algeria, specifically focusing on English in medicine, and identified several key issues. The findings revealed that inadequate preparation of teaching materials, a lack of well-trained ESP teachers, and insufficient motivation among students were the primary problems affecting the effectiveness of ESP instruction in this context. Addressing these challenges is crucial for improving the quality of English language education in medical fields in Algeria [17].

Exploring Teachers’ Perspectives on the Challenges of Teaching EMP in Algeria by Nachoua Kelkoula and Hanane Ouis uncovered several key findings. It showed that students recognized the significance of EMP but struggled to connect with the language. Furthermore, the research emphasized the necessity for various healthcare-related tasks and activities to improve the four essential language skills: listening, speaking, reading, and writing. These insights suggest that addressing these issues could enhance the effectiveness of EMP teaching in medical education [18].

### EMP in Iran

EMP is usually incorporated as a sequence of compulsory courses (6 credits) in the undergraduate medical curriculum in Iran during the pre-clinical years of the undergraduate medical program. The EMP curriculum mainly focuses on reading comprehension of medical texts and some basic medical vocabulary, with less focus on listening and speaking skills in a clinical environment.

There are few interactive or real materials, and the curriculum often stresses theoretical information over communication skills. The requirements for English language proficiency in medical school admissions have evolved substantially in recent years. Up until it was added to the national medical entrance examination (Konkur) in 2019, English served as a gatekeeper and motivator for medical students learning English. In 2020, English was taken out of the entrance exam for the medical and allied health programs, which had significantly diminished students’ desire to acquire English language proficiency before university. These policy changes will significantly affect EMP teaching and represent a need for new strategies to perhaps encourage students and to promote language development in the medical curriculum.

### Gamification

Gamification, defined as the application of game-design elements (e.g., points, badges, leaderboards) and principles in non-game contexts to enhance engagement and motivation, is an effective pedagogy in education [19]. This is supported by cogent theoretical frameworks, including Self-Determination Theory, which identifies autonomy, competence, and relatedness as key components contributing to or satisfying motivation; overall, gamification aligns with contemporary pedagogical approaches that prioritize active learning [20].

In medical education, gamified interventions have been found to be effective in improving knowledge retention, teamwork and learner satisfaction [21]. This study examined how gamification principles could be applied to address issues identified in medical English education, particularly in creating interactive and contextually relevant language practice.

Despite the existing body of research, there is a notable gap in qualitative studies that explore the specific challenges faced by medical educators and students in Iran regarding English language instruction. Most studies focus on quantitative assessments of language proficiency rather than the lived experiences and perceptions of those directly involved in the educational process.

This qualitative study explored these challenges through interviews with medical students and instructors. It examined their experiences and perceptions of effective strategies for the improvement of English language instruction in medical education. Furthermore, this also examined how these challenges can be addressed by leveraging technological tools to create a more interactive and engaging learning environment. The current study tried to add insight into the multifaceted dimensions of teaching medical English in Iran. It indicated what place integration of technology could take for significantly improving learning for future physicians. This exploration will lead to recommendations that can guide curriculum development and teaching methods, enabling medical students to be more effectively equipped to meet the challenges of a progressively interconnected world.

## Method

### Study design and setting

This qualitative thematic content analysis study explored the challenges of teaching English to medical students in Iran and was designed to ensure high standards of credibility, dependability, confirmability, and transferability [22] based on four criteria of Lincoln and Guba [23]. This study followed a multi-step approach in collecting data from respondents to ensure validity. Such an approach allowed for an in-depth inquiry into the exact nature of challenges in the specified setting. The research was conducted at medical universities across Iran, involving experienced medical English instructors and medical science students from various academic years. This setting provided a rich context for understanding the unique challenges within the medical education framework. The study was conducted from *September 2023 to February 2024.*

This study has been designed and written following the 32-item Consolidated Criteria for Reporting Qualitative Research (COREQ) checklist to ensure integrity and transparency. COREQ gives extensive guidance for integrity and transparency for qualitative research using interviews and focus groups. The COREQ checklist directed reporting around: researcher characteristics and reflexivity, study design, data collection, analysis, and findings. Details regarding the research team’s backgrounds and roles, participant selection, interview procedures, and data analysis are provided to ensure methodological transparency and reproducibility [24].

### Participants/ sampling

The study targeted a diverse group of participants, including instructors and students. The study involved participants from five medical universities across Iran, selected to represent diverse geographic and institutional contexts. Universities were chosen based on their active medical English programs and willingness to participate in the study.

Both instructors and students were recruited through purposive sampling to ensure participants had relevant experience with medical English education.

***Inclusion criteria for instructors*** included being faculty members with at least three years of experience teaching medical English and familiarity with modern educational methods and technologies.

The purposive sampling procedure started with five initial samples and eventually reached data saturation with 13 samples.

***The inclusion criteria for students*** included the non-guest medical university students from various academic years enrolled in medical English courses within the last three years at one of the selected universities. Visiting students from other universities and those who did not wish to continue with the interviews were excluded from the study. Student purposive sampling also started with five initial samples and reached saturation with 10 samples.

This method allowed for a broader perspective on the challenges encountered in medical English education. This diversity enhances the generalizability of the findings within the context of Iranian medical education. The selection of study participants continued until the saturation of data. Finally, a total of 23 (13 instructors and 10 students) were recruited for this study, balancing different educational backgrounds and experiences.

All participants were informed of the study and signed the informed consent form.

During the study period, students who were registered for the medical English course and officially enrolled in the university’s medical program were referred to as “non-guests.” To preserve uniformity in the curriculum exposure and educational context, visiting or guest students from other institutions were not allowed.

Students’ Grade Point Average (GPA)_ on a 0–20 scale, as commonly used in Iranian universities_ was gathered as required information on participants’ academic backgrounds, their academic standing in relation to their experiences learning medical English is framed by this measure.

### Tools/ instrument

The research instrument was a semi-structured questionnaire with 10 main questions and several probing questions, which were used during in-depth interview sessions. These questions underwent a thorough refinement process during the pilot phase. To increase the validity of the research, triangulation was employed, incorporating insights from experienced medical educators and research faculty at the medical university involved in the study, following the guidelines outlined in AMEE Guide No. 87, which emphasizes best practices in medical education research [25]. Additionally, a constructive approach was taken through cognitive interviews, where the researcher engaged in discussions with colleagues from the fields of public health and medical education.

The interview guide was developed by the research team for this specific study, based on prior literature and expert input. The semi-structured interview guides, developed separately for instructors and students, are provided in both English and Persian as Supplementary Files 1,2,3, and 4. These guides include core and probing questions to ensure consistency and depth across interviews. (see Supplementary Files 1,2,3 & 4)

***Interview Questions***.

1. What are the primary challenges you face in teaching English to medical students in Iran?
2. How do you perceive the effectiveness of current English language instruction for medical students?
3. Which language skills (listening, speaking, reading, writing) do you emphasize most in your classes, and why?
4. How do institutional policies and available resources affect your teaching of English in medical schools?
5. What infrastructural or organizational needs do you think are necessary for improving EMP instruction?
6. What strategies do you use to overcome language barriers among your students?
7. What role does technology play in facilitating or hindering English language learning for medical students?
8. In your experience, how does English proficiency impact students’ academic performance and clinical competencies?
9. How do you currently use technological tools in your courses? Are there any tools or platforms you find particularly effective?
10. What are your thoughts on incorporating gamification (using game-like elements) into EMP teaching? Have you used or considered such methods?

In addition to the main questions, which were conducted in a comfortable and open environment tailored to the conditions and choices of the participants, more detailed questions might have been asked to further explain the topic. Also, depending on the participants’ responses, follow-up questions were sometimes posed to elicit deeper answers.

### Data collection

#### Research team and reflexivity

The research team was made of four members: two with expertise in e-learning and medical education (ZZ, ZK), one with a background in qualitative research and English for Medical Purposes (NZ), and one with experience in teaching English as a foreign language (AF). The interviews were conducted by ZZ (Ph.D. candidate in e-learning), who had prior experience in qualitative interviewing and no direct teaching relationship with the participants, minimizing the chance of bias. Throughout the research process, the research team discussed coding strategies, worked together to create the interview guides, and regularly reflected on their positionality and assumptions throughout the research process.

#### Interviews

Semi-structured interviews were conducted in a quiet, private setting to create an environment where the discussion could be openly shared. Interviews were conducted either face-to-face or online (via Google Meet), depending on participant preference and considering geographical dispersion among the participants from different medical universities (Medical Universities of Iran).

Each interview lasted approximately 45 to 60 min and was audio-recorded with the participants’ approval. The interviews were then transcribed verbatim for analysis. The interviewer followed a semi-structured guide tailored to each participant group (instructors and students), allowing for flexibility to probe emerging topics. The interviewer maintained field notes to capture non-verbal cues and contextual factors.

Before interviews, participants received a brief explanation of gamification, including definition, application, and examples such as language-learning apps and scenario-based simulations. This guaranteed shared understanding while allowing participants to reflect on their experiences with or without being directly exposed to such methodologies.

To ensure comfort and clarity during data collection, all interviews and questionnaires were conducted in the participants’ native Persian (Farsi). Richer, more complex answers were made possible by this decision. Persian interview guides and questionnaires were created, and for analysis and reporting purposes, they were subsequently translated into English.

### Data analysis

Thematic content analysis was employed to analyze the transcribed interviews and transform raw qualitative data into meaningful insights in an iterative and dynamic approach.

Data analysis followed the steps outlined by Braun and Clarke for thematic analysis [26], including familiarization with the transcripts, coding by two independent researchers (ZZ, ZK), and iterative theme development through team discussions. Discrepancies in coding were resolved through consensus. To increase trustworthiness, member checking was performed by sharing preliminary findings with selected participants for feedback.

The analysis involved several steps:

- Familiarization with the Data: Repeated reading of interview transcripts and field notes to gain a deep understanding of the data.
- Identifying Significant Statements: Carefully selecting statements that captured the essence of participants’ experiences and generating preliminary descriptive labels for deeper analysis.
- Coding and Theme Development: Coding the data to identify significant themes related to the challenges faced by both instructors and students and categorizing these codes into broader themes that represent common issues in teaching and learning specialized English.
- Theme Review and Refinement: Reviewing themes to ensure they accurately represent the data in their most appropriate form.

Analysis was conducted based on the following definitions:

- Theme: Overarching conceptual category representing major research findings.
- Sub-themes: Secondary interpretive layer providing contextual understanding.
- Open Codes: Specific descriptive elements extracted directly from raw data.

In recording and transcribing the interviews, efforts were made to maintain the integrity of the text without altering the expression of sentences and phrases. After extracting themes, the interview transcripts were sent to participants for review and confirmation. Additionally, the themes and sub-themes were reviewed by three peers with PhDs in Medical Education and English Language Education. The analytical process involved continuous refinement of coding categories, creating a flexible framework. Researchers were receptive to emerging insights, repeatedly revisiting and adjusting initial codes based on consultation and peer feedback. This iterative approach provided a dynamic and comprehensive qualitative analysis that captured the complexity of the research phenomenon. In addition to qualitative content analysis, the frequency of each code was also counted. This method, which is considered quantitative content analysis, helps to consider the frequency and emphasis placed on each code.

### Reflexivity

Reflexivity was critical throughout the research process, as it involved reflecting on the researchers’ biases, experiences, and assumptions that could influence data collection and analysis. The frequent discussions between the research team allowed various perspectives to be considered, minimizing the probability of any single bias occurring. The researchers also mentioned their backgrounds in language education, which would influence how they viewed the data. This constant reflection helped the researchers be conscious of their perspectives and aware of how they may influence the data analysis.

### Trustworthiness

Some strategies were used to enhance trustworthiness, including member checking, which entailed sharing findings with participants to receive their feedback on the accuracy of interpretations, and triangulation, which consisted of comparing data from various participants, such as medical students and instructors. This approach ensured a complete understanding of the real challenges in learning and teaching medical English.

Audit trails were maintained during the data collection, analysis, and decision-making stages. Furthermore, peer debriefings were convened with colleagues unrelated to the study to share research findings and interpretations with them. All these increased the credibility and dependability of the study’s findings, which have provided useful insights into the many faces of English language education in medical contexts in Iran.

### Ethical considerations

This study received ethical approval from the Institutional Review Board of Shiraz University of Medical Sciences (IR.SUMS.REC.1403.510).

Before participation, all individuals had an information sheet with details of the study’s objectives, procedures, and possible harms and benefits. Written informed consent was obtained from each participant with an emphasis on their voluntary participation and ability to withdraw from participation at any time without repercussion. Participants were also given privacy and comfort, conducting interviews in the locations chosen by participants. During face-to-face interviews, a quiet and private room was secured prior to the interview at the university or an alternate location mutually agreed upon, to decrease interruptions and reasonably protect confidentiality.

Remote participants were offered a secure link to participate (Google Meet) while maintaining the ability to ensure a private consultation on both sides. Confidentiality was strictly maintained. All recordings and transcripts of the interviews were anonymized by removing identifying information and assigning each participant a unique code. All data (raw) was only accessible by the research team, and was stored securely on password protected devices. Audio and video recordings were completed for transcription analysis purposes only, and would be deleted after the study.

In discussing results, we were careful to report the data in aggregate form and not to disclose any information that could indirectly identify the participants. Participants were forewarned of the questions or nature of the questions and of the materials that would be audio or video recorded in advance of the interview. Each participant was emailed the interview guide in advance of the interview so they could review the subject matter and prepare for the interview. All the procedures were conducted under the Declaration of Helsinki and institutional policy guidelines.

## Results

The study involved 23 participants, including 13 medical English instructors aged between 45 and 58 and 10 medical students of Medical Universities in Iran aged between 19 and 38 whose demographic characteristics are depicted in Table 1. To contextualize students’ academic backgrounds, their GPA was recorded. GPA was reported on a 0–20 scale, which is standard in Iranian universities.

**Table 1:** Demographic information of the study participants

Instructors					
Participants Codes	Age	Gender	Job Experience	Educational level	
I01	58	Male	20	PhD	
I02	50	Female	15	PhD	
I03	51	Female	16	PhD	
I04	50	Female	15	PhD	
I05	48	Female	10	PhD	
I06	51	Female	16	PhD	
I07	51	Female	15	PhD	
I08	51	Male	14	PhD	
I09	50	Male	15	PhD	
I10	53	Male	14	PhD	
I11	49	Male	12	PhD	
I12	50	Male	10	PhD	
I13	45	Male	13	PhD	
Medical sciences students					
Participants Codes	Age	Gender	Semester	Major	GPA
S01	38	Female	6	Medical Education (PhD)	18.20
S02	38	Male	6	Medical Education (PhD)	19.34
S03	37	Male	7	eLearning (PhD)	16.80
S04	25	Female	10	MD student	17.54
S05	19	Male	4	MD student	17.84
S06	21	Male	6	MD student	15.68
S07	25	Male	8	MD student	16.26
S08	34	Female	4	eLearning(PhD)	18.83
S09	26	Female	9	MD student	17.56
S10	23	Female	7	MD student	19.03

*I: Instructor *S: Student *GPA: Grade Point Average, reported on a 0–20 scale

A total of 90 open codes were developed (50 open codes for challenges and 40 for recommendations), identifying 6 main themes and 25 subthemes (5 themes and 13 subthemes for challenges and 1 theme and 12 subthemes for recommendations).

These main themes were established, each containing unique subthemes and open codes that offered a deeper understanding of the complexities and challenges encountered by instructors and students (Table 2) and recommendations that were suggested by interviewees for improving English language education (Table 3).

**Table 2:** Thematic analysis of interviews with teachers and students about the challenges of english Language education

Themes (Main concepts)		Counts (*N*)	
Sub-themes (Components)	Open codes	Teachers (13)	Students (10)
Theme 1. Instructors			
Knowledge Gaps	1) Instructors’ insufficient medical terminology knowledge	6	4
	2) Shortage of qualified instructors who are proficient in both English and medical content	8	2
	3) Lack of confidence in teaching specialized content to medical students	5	4
	4) Debate over whether language instructors or subject specialists should teach medical English	7	5
Teaching Methodology	5) Traditional vs. modern teaching methods (focus on individual rather than cooperative)	11	8
	6) The methods are less interactive and lack feedback.	12	1
	7) Emphasis on translation and technical vocabulary at the expense of comprehensive language instruction	6	2
Integrating Technology	8) Challenges in integrating technology and innovation in medical language instruction	5	4
	9) Limitations in adapting new technologies in teaching	6	5
	10) Faculty resistance regarding the integration of technology in teaching	9	7
	11) Varying levels of technical proficiency among instructors	7	8
Innovative activities	12) Difficulty in motivating students	11	1
	13) Faculty resistance regarding innovative teaching approaches	1	6
Theme 2. Students			
Language Proficiency	14) Low proficiency in general English	10	2
	15) Pronunciation difficulties and unfamiliarity with medical terminology	8	6
	16) Lack of familiarity with medical terminology	9	4
	17) Inability to comprehend specialized texts due to foundational language weaknesses	7	1
	18) Varied language proficiency levels leading to unequal participation.	6	3
Attitude and perspective	19) Misconceptions about the role of language instructors	8	2
	20) Expectation for instructors to teach medical terminology	5	2
	21) Lack of engagement from other instructors in improving specialized language education	6	1
Interaction and participation	22) Limited opportunities for speaking practice in class due to large class sizes (over forty students)	12	8
	23) Anxiety about participating in English discussions	7	3
	24) Embarrassed to participate in class discussions for not being proficient	9	5
	25) Varied language proficiency levels among students lead to unequal participation in English class discussions	9	4
Theme 3. Curriculum & Content			
Content Relevance	26) Focus on reading comprehension over speaking and listening	5	10
	27) The use of outdated or irrelevant materials	2	7
	28) The use of different textbooks and materials by instructors leads to inconsistencies	6	6
	29) Emphasis on medical terminology without sufficient grammar instruction	7	3
	30) Unstructured delivery of course materials	2	6
Resource Availability	31) Limited access to supplementary learning materials(e.g., videos, exercises)	3	4
	32) Need for updated textbooks and resources	5	7
	33) Technical barriers related to access and usage of digital resources by students.	2	6
	34) Limited access to high-quality digital resources and teaching materials that are specifically tailored for medical English instruction	3	7
Theme 4. Organization			
Policy making and management	35) Lack of clear policy-making in the development of virtual education and appropriate guidelines	5	3
	36) Resistance of some policymakers and senior managers to technological changes	5	2
	37) Lack of established criteria for assessing the effectiveness of medical English instruction.	6	1
Rules and regulations	38) Declining language proficiency among students due to changes in entrance exam requirements^*^	7	2
	39) Instability in regulations and educational rules regarding the development of blended learning	8	2
	40) Lack of supportive and motivational regulations and rules in the development of blended learning	9	3
Theme 5. Technology-enhanced learning			
Infrastructure	41) Inconsistent or inadequate internet connectivity	3	1
	42) Lack of online platforms offering sufficient content customized to our medical English courses	5	2
	43) Lack of online platforms offering attractive and interactive approaches customized to our medical English course	8	4
	44) Need for technological tools to support asynchronous learning opportunities	7	5
Blended learning	45) The need for using the blended mobile-based method of teaching	8	5
	46) Need for using quicker teaching methods utilizing mobile	5	6
	47) Most blended classes are LMS-based, and other blended methods are not used	9	3
	48) Insufficient training for instructors on how to design and implement blended learning courses (Use of technology)	7	2
	49) Balancing the demands of online and face-to-face components can be challenging for students (potential overload/stress).	6	5
	50) Using online and face-to-face classes is time-consuming	7	6

To extract themes and sub-themes, each interview text was analyzed through a precise and iterative back-and-forth process. Initially, codes were extracted inductively, and then components and themes were identified based on the meaning and content of each matter. Some quotes related to participants’ challenges in interviews are given:

### Theme 1- instructors

Sub Theme-Knowledge Gaps

*" I am not trained in medical terminology*,* so I try to learn enough to stay ahead of the students*,* but it’s challenging……. finding someone who is truly bilingual and also understands the complexities of medicine is like finding a needle in a haystack…” (Instructor 3).* And *“I think it should be a team*,* a language teacher with a medical doctor. That way*,* the students get the best of both worlds.” (Student 6)*.

Sub Theme-Teaching Methodology

*“I wish there were more opportunities to use the language in class*,* instead of just memorizing lists of words…….” (Student 9)*.

Sub Theme-Integrating Technology.

*“Some of my colleagues are just not interested in using computers or online tools.” (Instructor 4)*.

### Sub Theme-Innovative activities

*“It is really really hard to engage the learners in activities or do something different…… I’m not sure it is worth it to try and incorporate new teaching methods…….”. (Instructor 1)*

### Theme 2. Students

#### Sub Theme-Language proficiency

*“Pronunciation is a big problem……. I know the words*,* but I can’t say them correctly” (Student 6). And “When I read the text*,* there are many new words and terms that I have never seen before” (Student 4)*.

#### Sub Theme- attitude and perspective

*“I really believe that English teachers should participate more and give new tips in specialized language.” (Student 1)*.

#### Sub Theme- interaction and participation

*“There are too many students in our class*,* almost 40*,* so there is no chance for me to speak……. if there was any opportunity I feel that I’m afraid of making mistakes and looking stupid” (Student 7)*.

### Theme 3- curriculum & content

#### Sub Theme-Content relevance

*“We do all the reading in class*,* but we have to practice talking” (Student 2). And “We learn all these medical terms*,* but we don’t know how to put them in a sentence……. The teacher jumps from one thing to another with no organization……. " (Student 8)*.

#### Sub Theme-Resource availability

*“The books are outdated and don’t cover current medical practices; we need new and updated ones……. I don’t have access to online materials” (Student 9)*.

### Theme 4. Organization

#### Sub Theme- policy making and management

*“The administrators don’t care about improving technology*,* they don’t want to change……. We don’t know if what we are doing is working or not……. " (Instructor 5)*.

#### Sub Theme- rules and regulations

*“Because of the new rules in entrance exam and removing English from the entrance exam tests*,* students English knowledge has gotten worse…………….Everything changes all the time*,* regulations change a lot*,* so you are never sure what will happen….” (Instructor 8)*.

### Theme 5. Technology-enhanced learning

#### Sub Theme-Infrastructure

*“I can’t find suitable content that is made for medical course……. We need content that will engage the students” (Instructor 3)*.

#### Sub Theme- blended learning

*“I think it should be a phone-based app*,* the student always has their phones………It’s tough to do everything at once….” (Student) “*.

**Table 3:** Thematic analysis of interviews with instructors and students about recommendations for improvement of english Language education

Theme 1. Recommendations		Teachers	Students
Infrastructure Needs	51) Strengthening English language foundations for both students and instructors	10	7
	52) Implement joint teaching by subject specialists and English language instructors	6	10
	53) Integration of Gamification Strategies in Curriculum Development	11	10
	54) Create tailored courses that focus specifically on medical terminology, patient communication, and professional writing skills relevant to healthcare settings	6	10
Overcoming Barriers	55) Leverage technology to create interactive and engaging learning experiences	5	10
	56) Need for specialized training for instructors in the medical subject	4	7
	57) Providing both students and instructors access to high-quality resources	10	5
	58) Encourage faculty and students to engage in research related to medical English education, fostering innovation and the development of best practices that can address existing barriers	5	5
Skill Emphasis in Specialized Language Classes	59) Providing writing skills notes emphasized for certain courses alongside specialized terminology	4	6
	60) Focus on all four language skills (reading, writing, speaking, listening) but with varying emphasis depending on the course and student needs	4	6
Student Preferences	61) Training and courses should be planned to train teachers who are both medical teachers and language teachers		
Importance of English Language Skills	62) Focus on writing and reading English essential for accessing medical knowledge and publishing.	9	10
	63) Influence on communication English skills for clinical practice and patient interaction	7	10
Motivation	64) Recognition that motivation plays a crucial role in mastering English related to professional success.	5	6
	65) Understanding the practical applications of English proficiency in future careers	5	6
	66) Implement strategies to enhance student motivation, such as gamification, real-world applications of medical English, and opportunities for practical experience in clinical settings	6	10
	67) Use formative assessments to gauge student progress regularly and provide constructive feedback that helps them improve their language skills over time.	7	9
Academic Performance Correlation	68) Impact of English proficiency on academic performance in medical courses.	9	5
	69) Influence of language skills on clinical competence and patient interactions	6	3
Online learning platform	70) Provide accessible online platforms for language practice	4	10
	71) Provide a platform including multimedia, videos, animations, and e-content for teaching medical English	5	9
	72) Provide a thematic approach to course design with online learning platforms for teaching medical	6	6
Personalize learning	73) Provide a personal environment suitable for students’ Medical English level	6	10
	74) Need for conditions where students with different abilities can learn at their own pace	7	10
	75) Creating conditions where learning occurs without direct supervision and others’ judgment	5	9
	76) Provide opportunities for self-reflection and improvement based on gamification and interaction	6	9
Innovation and fun	77) Increase student participation through fun and gamified activities	6	10
	78) Use of rewards and incentives to motivate learners	7	9
	79) Creation of a fun and interactive learning environment	8	10
	80) Provide immediate feedback mechanisms integrated into gamified activities	9	10
	82) Assessment of language skills through interactive and gamified activities	9	6
	84) Attractive micro an fun content	4	5
Technology integration	85) Offer technical support and training to students on how to access and use digital resources.	3	4
	86) Invest in the development or acquisition of high-quality digital resources that are specifically designed for medical English instruction.	6	2
	87) Develop a centralized digital resource repository with recorded lectures, interactive exercises, and self-assessment tools to facilitate asynchronous learning.	5	6
	88) Adapt existing online resources to be mobile-friendly, ensuring accessibility on smartphones and tablet	2	7
	89) Provide training for instructors on designing and implementing blended learning courses	9	5
	90) Develop standardized assessment criteria that evaluate various aspects of medical English proficiency	10	5
Rules and regulations	91) Develop clear policies and guidelines for virtual education and blended learning.	3	5
	93) Establish supportive and motivational regulations for blended learning.	7	3

### Recommendations

Some quotes related to participants’ recommendations in interviews are given:

#### Sub Theme-Infrastructure needs

*“To be able to effectively teach Medical English*,* there is a necessity to reinforce the English language abilities of our instructors. Although they do their best*,* the constraint is that they are not familiar with medical terms.…….” (Instructor 6). And “I think having both a language teacher and a medical expert in the classroom would be ideal. That way*,*……. we get the best of both sections.” (Student 2)*.

#### Sub Theme-Overcoming barriers

*“As a teacher*,* I know that my lack of confidence in teaching specialized medical content is because of a lack of training.…To excel and provide the best learning experience to my students*,* I need access to specialized training in medical subjects. This will boost my confidence*,* and I can provide accurate*,* engaging*,* and effective lessons in Medical English.” (Instructor 1). And “The books we use are old and not current with the medical practices…. It’s hard to keep the students interested when the material isn’t relevant.” (Instructor 6)*.

#### Sub Theme- skill emphasis in specialized Language classes

*“We do so much translation of texts and learning technical words by heart*,* but we never get to practice speaking or listening…. How are we supposed to communicate with patients if we don’t practice?” (Student 2). And “We do so much reading and translating texts*,* but we never get to practice speaking or listening. How are we supposed to communicate with patients if we don’t practice?” (Student 9)*.

#### Sub Theme-Student preferences

*“I think it should be a team*,* a language teacher with a medical doctor. That way*,* the students get the best of both worlds” (Student 5)*.

#### Sub Theme- importance of english Language skills

*“Proficiency in reading and writing English is not just an academic skill; it is the gateway to accessing global medical literature and contributing to scientific discourse” (Instructor 7)*.

#### Sub Theme-Motivation

*“…. we see the possibilities of our students*,* but we have no interactive methods with supportive feedback which will increase their motivation. In order to revive once more their desire to study Medical English*,* we need to employ other measures*,* we can utilize gamification*,* real-world application*,* and practice in clinics. These tools enhance learning interest and helping driven and connect with their goals.” (Instructor 10). And “If there were interactive activities in the classroom*,* like games or role-playing*,* I think learning medical English would be more enjoyable and less stressful……. " (Student 10)*.

#### Sub Theme-Academic performance correlation

*“Language skills are not just academic tools; they are clinical tools that shape a student’s ability to diagnose*,* treat*,* and empathize with patients effectively” (Instructor 4)*.

#### Sub Theme-Online learning platform

*“……. If we had access to online platforms where we could practice speaking and get instant feedback…… it would make learning much more engaging….” (Student 8). And “……. We need more interactive content*,* like videos and quizzes*,* to make learning more engaging” (Student 9)*.

#### Sub Theme-Personalize learning

*“……. It’s hard to practice speaking in a class of 40 students. We need smaller groups or more interactive activities.” (Student 14). And “In large classes*,* students with lower proficiency often get left behind……. We need to create smaller*,* more focused groups to give everyone a chance to speak.” (Instructor 9)*.

#### Sub Theme- innovation and fun

*“Gamification could be a great way to make the lessons more interactive. For example*,* we could create a medical terminology game where students compete to answer questions.” (Instructor 6)*.

#### Sub Theme-Technology integration

*“Some of our English teachers struggle to teach medical terms properly……. It’s frustrating because we need to learn this so that we can work in the future.” (Student 7). And “We need more training on how to use technology as a component of teaching. Many of us are not experienced with tools like online platforms or gamification…….” (Instructor 7)*.

#### Sub Theme-Rules and regulations

*“It’s hard to know how to best use these online tools when there aren’t clear guidelines or expectations.……. Do we have to record our lectures?……. How do we handle student questions online? It would be helpful to have some clear policies to follow.” (Instructor 3)*.

## Discussion

This qualitative study presents a rich view of the challenges encountered in medical English universities in Iran, providing insightful details on the barriers to inhibit effective teaching and learning. The findings reveal a complex interplay of factors, encompassing instructor-related issues (knowledge gaps, teaching methodology, technology integration), student-related issues (language proficiency, attitudes, participation), curriculum and content issues (relevance, resources), organizational issues (policy, regulations), and technology-related issues (infrastructure, blended learning).

The **knowledge gaps** recognized among instructors, particularly regarding specialized medical terminology, are consistent with findings from studies conducted by Hani Elgindi et al. (2022) in Saudi Arabia [15], pointing to a general need for targeted professional development programs. Elgindi’s study specifically highlighted the difficulties new teachers face in adjusting English words to the medical context, managing pronunciation challenges, and comprehending intricate medical terminology. This is reinforced by instructors in the current study, who were not adequately trained to teach specialized content to medical students. In addition, Anthony’s (2018) [27] research in Southeast Asia, where ESP instructors reported similar challenges in mastering domain-specific vocabulary, revealed that terminology gaps among non-specialist instructors undermine pedagogical effectiveness, necessitating interdisciplinary collaboration. Our results extend this observation to Iran, suggesting that even instructors with medical backgrounds face challenges due to rapid advancements in biomedical terminology.

Moreover, the current study reveals an interesting **debate regarding the optimal approach to teaching medical English**, whether it should be primarily handled by language instructors or subject matter specialists. Some students expressed a preference for instructors with expertise in medical terminology, while others emphasized the importance of strong pedagogical skills and language expertise. This debate is consistent with discussions in the broader field of ESP, where the ideal balance between subject matter knowledge and language teaching expertise remains a topic of ongoing negotiations [28, 29]. The findings also explain the persistence of traditional teaching methods, with their focus on individual work and limited interactive opportunities. Many students expressed a desire for more engaging and communicative activities, showing concerns raised by Yang et al. [30]in their study on EMP instruction in China. Yang et al. emphasized the importance of effective self- and peer-learning strategies, which align with the current study’s findings regarding the need for more student-centered and interactive learning approaches.

Another finding of this study on **Student Language Proficiency and Engagement** highlighted students’ struggles with general English. This aligns with the findings of Ghimire’s study, where medical students’ limited general English proficiency set back their ability to comprehend clinical case studies [31]. Also, the findings of the present research on the challenges of specialized English **(**participants’ struggles with pronunciation and comprehension) mirrors Pandy and colleagues’ study [32], where students’ limited exposure to authentic clinical English interactions hindered fluency.

Also, the result of this study on **Curriculum Relevance and Resource Scarcity** and the emphasis on outdated textbooks and unstructured materials reflect more issues within ESP curricula. In this regard, Hyland’s (2016) [33] framework for needs analysis in ESP underlines the importance of arranging materials with learners’ professional contexts—a gap that the participants identified in our study. The participant’s confusion on how to use medical words in a sentence echoed Hyland’s critique of “decontextualized vocabulary drills”. The results of this research on the **importance of curriculum relevance** and resource availability are also consistent with those of Basturkmen (2021) [34], who emphasized the importance of fulfilling students’ needs and providing relevant discourse communities in ESP courses.

Another result of this study on **Organizational Resistance and Policy Gaps** regarding the resistance to technology among Iranian policymakers is similar to findings from Alvarez’s study [35] on educational reforms, where institutional inertia prevents blended learning initiatives. This research highlights how institutional inertia, including policy gaps and insufficient support systems, can obstruct blended learning reforms. However, our participants uniquely tied this resistance to regulatory instability (frequent changes in entrance exam requirements), an under-researched issue in existing EMP literature.

The results of the present study on the **Technology Integration and Blended Learning** regarding faculty resistance to technology contrast with optimistic accounts of EdTech adoption in Global North contexts. Yang’s study [36] reported the strong interest of instructors for virtual reality in EMP, but the results of our study revealed that technical proficiency gaps and limited institutional support obstruct similar enthusiasm in Iran. In addition, our participants’ request for mobile-based learning is consistent with Chung’s research results in South Korea [37], where WhatsApp-mediated micro-learning modules improved medical students’ situational language use. However, their success relied on South Korea’s high smartphone penetration and digital infrastructure. Also, the findings of this study on the lack of technological infrastructure are consistent with the results of Kelkoula and Ouis (2022) [18] on EMP in Algeria, which highlighted the necessity for enhanced technological infrastructure and adequate training for instructors.

The study revealed resistance to technology among **policymakers and senior managers**. The findings of this study demonstrated the necessity of established criteria for assessing the effectiveness of medical English instruction. These findings are similar to a systematic review by Macaro et al. [38], noting inconsistent definitions of language proficiency across studies and a lack of consensus on assessment methodologies, and necessitates the development of criteria for assessing the effectiveness of medical English instruction.

### Suggestions for future research

Due to the qualitative nature and the small sample size of this study, future research may use mixed-methods or a longitudinal design to examine the impact of technologies and gamification on EMP learning outcomes across educational contexts in Iran. Comparatively researching the EMP curriculum and educational system in other non-English speaking countries would be helpful for cross-cultural comparison. Future research may also study the viewpoints of policy-makers, curriculum developers, and clinical faculty to detail systemic barriers and facilitators for an effective integration of EMPs. Lastly, it would be helpful to conduct intervention studies to investigate the effectiveness of specific gamified or technology-enhanced EMP modules for evidence-based curriculum reform.

This study advances the EMP literature by highlighting regulatory instability as a compounding challenge in non-Western contexts. Identifying class size as a critical yet overlooked barrier to interactive language practice. Proposing gamification as a culturally adaptable strategy for low-resource settings.

Future studies could focus on the development and evaluation of specific technology-supported learning modules, blended learning patterns, and teacher training programs that are culturally appropriate for the Iranian medical education system. Also, there is a necessity to design engaging blended or online programs for students and instructors. This may need to be performed with an interdisciplinary team to ensure quality. Also, one should plan to create blended medical and English studies to ensure better opportunities for students.

### Strength and limitation

The study employed a comprehensive methodology, utilizing in-depth interviews with specialized English language teachers and students to explore challenges in English language education. It aimed to provide valuable insights for educational planners. Diverse samples were selected to ensure varied perspectives; however, certain limitations are inherent. As a qualitative study, its findings may not be universally applicable across broader populations or different educational systems, as challenges can differ significantly by region and demographic context. Additionally, the research was conducted in Iranian medical universities, where English is a secondary academic language. This context limits the applicability of results to institutions where English is the native language, as outcomes might vary substantially. Students’ proficiency levels in English also influence their experiences, further complicating generalization. Overall, these factors highlight the need for caution when interpreting and applying the study’s findings to different contexts.

## Conclusion

This study provides an elaborate description of the intricate matters at stake in medical English education in the Iranian context. The results of this study demonstrate the need for a unified and integrated approach to the provision of medical English education, encompassing better instructor preparation and interprofessional collaboration, interactive learning spaces, relevant curriculum development, firm organizational support, and the cautious application of technology. To facilitate more productive and effective learning experiences, there should be a community of collaboration where subject experts and language instructors collaborate to create more engaging experiences for students. There must be high student motivation so that they are encouraged to present their best efforts. These recommendations, when implemented systematically and thoughtfully, can significantly enhance the standard of medical English education in Iran, equipping future physicians with the language to thrive in an increasingly integrated world healthcare setting. It will render them more confident as they communicate in discussions with medical instructors, staff, and patients. To enhance the level of medical English in Iran, it is recommended to integrate technology and innovation in medical English, with a particular focus on addressing instructors’ needs, such as practical skills, and providing peer-to-peer learning opportunities. Care should also be taken to use modern resources such as online journals and animated videos.

## Electronic supplementary material

Below is the link to the electronic supplementary material.

Supplementary Material 1

Supplementary Material 2

Supplementary Material 3

Supplementary Material 4

## Data Availability

The data analyzed during the current study are available from the corresponding author upon reasonable request.

## Abbreviations

EMP: English for Medical Purposes
ESP: English for Specific Purposes
