# Supplementary material for: Navigating challenges in medical english learning: leveraging technology and gamification for interactive education – a qualitative study
Source: BMC Med Educ. 2025 Jul 12;25:1045. doi: 10.1186/s12909-025-07511-1 (PMC12255984; doi:10.1186/s12909-025-07511-1)
Supplement: Supplementary file 1 — Supplementary Material 1 [file 12909_2025_7511_MOESM1_ESM.docx]

**Supplementary File 1 – Semi-Structured Interview Guide for Instructors (English Version)**

**Title of Study:** Navigating Challenges in Medical English Learning: Leveraging Technology and Gamification for Interactive Education

**Purpose of Interview**

To explore the experiences and perceptions of medical instructors and students regarding the challenges and potential solutions in Medical English education in Iran, with a focus on the role of technology and gamification.

**Participant Information**

- Name: _____________________________________
- Gender _____________________________________
- Age________________________________________
- Degree / Field of Study________________________
- Job Experience (years) ________________________
- Institution: _________________________________
- Participant Codes (matching the audio file) ________
- Audio File Number:  _________________________
- Date of Interview: ___________________________

| **Title of Study** | Navigating Challenges in Medical English Learning: Leveraging Technology and Gamification for Interactive Education – A Qualitative Study |
| --- | --- |
| **Introduction** | Thank you for agreeing to participate in this interview. The purpose is to explore your experiences and perspectives on teaching English for Medical Purposes (EMP) in Iran, with a focus on challenges, technology, and gamification. |
| **Main Interview Questions** | 1. What are the primary challenges you face in teaching English to medical students in Iran? |
|  | 1. How do you perceive the effectiveness of current English language instruction for medical students? |
|  | 1. Which language skills (listening, speaking, reading, writing) do you emphasize most in your classes, and why? |
|  | 1. How do institutional policies and available resources affect your teaching of English in medical schools? |
|  | 1. What infrastructural or organizational needs do you think are necessary for improving EMP instruction? |
|  | 1. What strategies do you use to overcome language barriers among your students? |
|  | 1. What role does technology play in facilitating or hindering English language learning for medical students? |
|  | 1. In your experience, how does English proficiency impact students’ academic performance and clinical competencies? |
|  | 1. How do you currently use technological tools in your courses? Are there any tools or platforms you find particularly effective? |
|  | 1. What are your thoughts on incorporating gamification (using game-like elements) into EMP teaching? Have you used or considered such methods? |
| **Probing Questions**  **(used when needed)** | - Can you give an example? |
|  | - How did that impact your teaching? |
|  | - What alternatives do you think would work better? |
|  | - How do you suggest that the issue could be improved? |
|  | - How did you address this challenge? |
|  | - How do students respond to these approaches? |

**Additional Notes/Comments**

|  |
| --- |
