# Supplementary material for: Navigating challenges in medical english learning: leveraging technology and gamification for interactive education – a qualitative study
Source: BMC Med Educ. 2025 Jul 12;25:1045. doi: 10.1186/s12909-025-07511-1 (PMC12255984; doi:10.1186/s12909-025-07511-1)
Supplement: Supplementary file 2 — Supplementary Material 2 [file 12909_2025_7511_MOESM2_ESM.docx]

**Supplementary File 2 – Semi-Structured Interview Guide for Students (English Version)**

**Title of Study:** Navigating Challenges in Medical English Learning: Leveraging Technology and Gamification for Interactive Education

**Purpose of Interview**

To explore the experiences and perceptions of medical instructors and students regarding the challenges and potential solutions in Medical English education in Iran, with a focus on the role of technology and gamification.

**Participant Information**

- Name: _____________________________________
- Gender _____________________________________
- Age________________________________________
- Degree / Field of Study________________________
- Institution: _________________________________
- Participant Codes (matching the audio file) ________
- Audio File Number:  _________________________
- Date of Interview: ___________________________
- Semester: __________________________________
- GPA: _____________________________________

| **Title of Study** | Navigating Challenges in Medical English Learning: Leveraging Technology and Gamification for Interactive Education – A Qualitative Study |
| --- | --- |
| **Introduction** | Thank you for participating in this interview. We would like to learn about your experiences with learning English for Medical Purposes (EMP), including challenges, the use of technology, and your views on gamification. |
| **Main Interview Questions** | 1. What challenges have you experienced in learning English for medical purposes? |
|  | 1. How effective do you find the current English language instruction in your medical program? |
|  | 1. Which language skills do you find most difficult (listening, speaking, reading, writing), and why? |
|  | 1. How do university policies and resources affect your English learning experience? |
|  | 1. What improvements do you think are needed in the EMP curriculum or teaching methods? |
|  | 1. What strategies do you use personally to overcome difficulties in medical English? |
|  | 1. What role does technology play in your English learning? Are there specific tools or platforms you use? |
|  | 1. How does your proficiency in English affect your academic performance and clinical training? |
|  | 1. Have you experienced any technology-enhanced or interactive learning activities in your English courses? If yes, please describe. |
|  | 1. What do you think about using gamification (game-like elements) in learning medical English? Would you be interested in such methods? |
| **Probing Questions**  **(used when needed)** | - Can you describe a specific situation? |
|  | - What helped you most? |
|  | - How did you feel about this approach? |
|  | - Would you recommend any changes? |

**Additional Notes/Comments**

|  |
| --- |
