# Supplementary material for: Navigating challenges in medical english learning: leveraging technology and gamification for interactive education – a qualitative study
Source: BMC Med Educ. 2025 Jul 12;25:1045. doi: 10.1186/s12909-025-07511-1 (PMC12255984; doi:10.1186/s12909-025-07511-1)
Supplement: Supplementary file 3 — Supplementary Material 3 [file 12909_2025_7511_MOESM3_ESM.docx]

**راهنمای مصاحبه نیمه‌ساختاریافته برای اساتید زبان انگلیسی تخصصی پزشکی**

**عنوان مطالعه:** پیمایش چالش‌ها در یادگیری زبان انگلیسی پزشکی: بهره‌گیری از فناوری و بازی‌وارسازی برای آموزش تعاملی

**هدف مصاحبه:** بررسی تجربیات و دیدگاه‌های اساتید و دانشجویان پزشکی درباره چالش‌ها و راهکارهای احتمالی در آموزش زبان انگلیسی پزشکی در ایران، با تمرکز بر نقش فناوری و بازی‌وارسازی.

**اطلاعات شرکت‌کننده**

- **نام_____________________________________**
- **جنسیت__________________________________**
- **سن_____________________________________**
- **مدرک / رشته تحصیلی_______________________**
- **سابقه شغلی (سال_________________________**
- **مؤسسه__________________________________**
- **کد شرکت‌کننده (مطابق با فایل صوتی_________**
- **شماره فایل صوتی_________________________**
- **تاریخ مصاحبه_____________________________**

| پیمایش چالش‌ها در یادگیری زبان انگلیسی پزشکی: بهره‌گیری از فناوری و بازی‌وارسازی برای آموزش تعاملی | **عنوان مطالعه** |
| --- | --- |
| از اینکه در این مصاحبه شرکت می‌کنید سپاسگزاریم. هدف این مصاحبه بررسی تجربیات و دیدگاه‌های شما در زمینه آموزش زبان انگلیسی برای اهداف پزشکی در ایران، با تمرکز بر چالش‌ها، فناوری و بازی‌وارسازی است | **مقدمه** |
| 1. مهم‌ترین چالش‌هایی که در آموزش زبان انگلیسی به دانشجویان پزشکی در ایران با آن مواجه هستید چیست؟ | **سوالات اصلی** |
| 1. اثربخشی آموزش زبان انگلیسی فعلی برای دانشجویان پزشکی را چگونه ارزیابی می‌کنید؟ |  |
| 1. کدام مهارت‌های زبانی (شنیداری، گفتاری، خواندن، نوشتن) را در کلاس‌های خود بیشتر مورد تأکید قرار می‌دهید و چرا؟ |  |
| 1. سیاست‌ها و منابع موجود در دانشگاه تا چه اندازه بر آموزش زبان انگلیسی شما تأثیرگذار است؟ |  |
| 1. به نظر شما چه نیازهای زیرساختی یا سازمانی برای بهبود آموزش زبان انگلیسی پزشکی لازم است؟ |  |
| 1. برای غلبه بر موانع زبانی دانشجویان خود از چه راهبردهایی استفاده می‌کنید؟ |  |
| 1. نقش فناوری را در تسهیل یا مانع‌تراشی یادگیری زبان انگلیسی برای دانشجویان پزشکی چگونه می‌بینید؟ |  |
| 1. از نظر شما، تسلط به زبان انگلیسی چه تأثیری بر عملکرد تحصیلی و مهارت‌های بالینی دانشجویان دارد؟ |  |
| 1. در حال حاضر از چه ابزارهای فناورانه‌ای در کلاس‌های خود استفاده می‌کنید؟/ آیا پلتفرم یا ابزاری هست که اثربخشی خاصی داشته باشد؟ |  |
| 1. نظر شما درباره استفاده از بازی‌وارسازی (استفاده از عناصر بازی) در آموزش زبان انگلیسی پزشکی چیست؟ آیا تا به حال از این روش‌ها استفاده کرده‌اید یا به آن فکر کرده‌اید؟ |  |
| می‌توانید مثالی بزنید؟ | **سوالات پیگیری** |
| این موضوع چه تأثیری بر تدریس شما داشت؟ |  |
| فکر می‌کنید چه جایگزین‌هایی می‌توانند بهتر عمل کنند؟ |  |
| چه پیشنهادی برای بهبود این مسئله دارید؟ |  |
| چگونه با این چالش برخورد کردید؟ |  |
| واکنش دانشجویان به این رویکردها چگونه بود؟ |  |

**سایر نظرات**

|  |
| --- |
