# Supplementary material for: Navigating challenges in medical english learning: leveraging technology and gamification for interactive education – a qualitative study
Source: BMC Med Educ. 2025 Jul 12;25:1045. doi: 10.1186/s12909-025-07511-1 (PMC12255984; doi:10.1186/s12909-025-07511-1)
Supplement: Supplementary file 4 — Supplementary Material 4 [file 12909_2025_7511_MOESM4_ESM.docx]

**راهنمای مصاحبه نیمه‌ساختاریافته برای دانشجویان پزشکی**

**عنوان مطالعه:** پیمایش چالش‌ها در یادگیری زبان انگلیسی پزشکی: بهره‌گیری از فناوری و بازی‌وارسازی برای آموزش تعاملی

**هدف مصاحبه:** بررسی تجربیات و دیدگاه‌های اساتید و دانشجویان پزشکی درباره چالش‌ها و راهکارهای احتمالی در آموزش زبان انگلیسی پزشکی در ایران، با تمرکز بر نقش فناوری و بازی‌وارسازی.

**اطلاعات شرکت‌کننده**

- **نام_____________________________________**
- **جنسیت__________________________________**
- **سن_____________________________________**
- **مدرک / رشته تحصیلی_______________________**
- **مؤسسه__________________________________**
- **کد شرکت‌کننده (مطابق با فایل صوتی______________**
- **شماره فایل صوتی___________________________**
- **تاریخ مصاحبه______________________________**
- **ترم تحصیلی _____________________________**
- **معدل ___________________________________**

| پیمایش چالش‌ها در یادگیری زبان انگلیسی پزشکی: بهره‌گیری از فناوری و بازی‌وارسازی برای آموزش تعاملی | **عنوان مطالعه** |
| --- | --- |
| از اینکه در این مصاحبه شرکت می‌کنید سپاسگزاریم. هدف این مصاحبه بررسی تجربیات شما در یادگیری زبان انگلیسی برای اهداف پزشکی، از جمله چالش‌ها، استفاده از فناوری و دیدگاه شما نسبت به بازی‌وارسازی است. | **مقدمه** |
| 1. چه چالش‌هایی در یادگیری زبان انگلیسی پزشکی تجربه کرده‌اید؟ | **سوالات اصلی** |
| 1. آموزش زبان انگلیسی فعلی در برنامه پزشکی خود را تا چه حد مؤثر می‌دانید؟ |  |
| 1. کدام مهارت‌های زبانی (شنیداری، گفتاری، خواندن، نوشتن) برای شما دشوارتر است و چرا؟ |  |
| 1. سیاست‌ها و منابع دانشگاه چه تأثیری بر تجربه یادگیری زبان انگلیسی شما دارد؟ |  |
| 1. چه بهبودهایی را در برنامه درسی یا روش‌های آموزش زبان انگلیسی پزشکی لازم می‌دانید؟ |  |
| 1. برای غلبه بر مشکلات یادگیری زبان انگلیسی پزشکی، شخصاً از چه راهبردهایی استفاده می‌کنید؟ |  |
| 1. نقش فناوری را در یادگیری زبان انگلیسی خود چگونه می‌بینید؟ آیا از ابزار یا پلتفرم خاصی استفاده می‌کنید؟ |  |
| 1. تسلط شما به زبان انگلیسی چه تأثیری بر عملکرد تحصیلی و آموزش بالینی شما دارد؟ |  |
| 1. آیا تا به حال در کلاس‌های زبان انگلیسی خود تجربه فعالیت‌های تعاملی یا مبتنی بر فناوری داشته‌اید؟ اگر بله، لطفاً توضیح دهید |  |
| 1. نظر شما درباره استفاده از بازی‌وارسازی (عناصر بازی) در یادگیری زبان انگلیسی پزشکی چیست؟ آیا به استفاده از این روش‌ها علاقه‌مند هستید؟ |  |
| می‌توانید یک موقعیت خاص را توصیف کنید؟ | **سوالات پیگیری** |
| چه چیزی بیشتر به شما کمک کرد؟ |  |
| نظرتان درباره این رویکرد چه بود؟ |  |
| آیا پیشنهادی برای تغییر دارید؟ |  |

**سایر نظرات**

|  |
| --- |
